# What do we talk about when we talk about “equipoise”? Stakeholder interviews assessing the use of equipoise in clinical research ethics

**DOI:** 10.1186/s13063-023-07221-3

**Published:** 2023-03-18

**Authors:** Brian Dewar, Stephanie Chevrier, Julie De Meulemeester, Mark Fedyk, Rosendo Rodriguez, Simon Kitto, Raphael Saginur, Michel Shamy

**Affiliations:** 1 Ottawa Hospital Research Institute, Ottawa, Canada; 2 University of California, Davis, Davis USA; 3 Department of Medicine, University of Ottawa, Ottawa, Canada; 4 Department of Innovation in Medical Education, University of Ottawa, Ottawa, Canada

**Keywords:** Ethics, Clinical trials, Research, Equipoise

## Abstract

**Introduction:**

Equipoise, generally defined as uncertainty about the relative effects of the treatments being compared in a trial, is frequently referenced as an ethical standard for the conduct of randomized clinical trials. However, it seems to be defined in several different ways and may be used differently by different individuals. We explored how clinical researchers, chairs of research ethics boards, and philosophers of science define and reason with this term.

**Methods:**

We completed semi-structured interviews about clinical trial ethics with 15 clinical researchers, 15 research ethics board chairs, and 15 philosophers of science/bioethicists. Each participant was asked a standardized set of 10 questions, 4 of which were specifically about equipoise. All interviews were conducted telephonically and transcribed. Responses were grouped and analysed via a modified grounded theory method.

**Results:**

Forty-three respondents defined equipoise in 7 logically distinct ways, and 2 respondents could not explicitly define it. The most common definition, offered by 14 respondents (31%), defined “equipoise” as a disagreement at the level of a community of physicians. There was significant variability in definitions offered between and within groups. When asked how they would “operationalize” equipoise — i.e. check or test for its presence — respondents provided 7 alternatives, the most common being in relation to a literature review (15/45, 33%). The vast majority of respondents (35/45, 78%) felt the concept was helpful, though many acknowledged that the lack of a clear definition or operationalization was problematic.

**Conclusion:**

There is significant variation in definitions of equipoise offered by respondents, suggesting that parties within groups and between groups may be referring to different concepts when they reference “equipoise”. This non-uniformity may impact fairness and transparency and opens the door to potential ethical problems in the evaluation of clinical trials — for instance, a patient may understand equipoise very differently than the researchers enrolling her in a trial, which could cause her agreement to participate to be based upon false premises.

**Supplementary Information:**

The online version contains supplementary material available at 10.1186/s13063-023-07221-3.

## Key points

Question: How do clinical researchers, ethics regulators, and philosophers of science understand and implement the term “equipoise”?

Findings: In this series of interviews of 15 clinical trialists, 15 ethics regulators, and 15 philosophers of science, equipoise was defined 7 different ways with significant variability in responses offered between and within groups of respondents.

Meaning: The variation in definitions of equipoise offered suggests that parties within groups and between groups may be referring to different concepts when utilizing the term “equipoise”, creating the potential for ethical problems.

## Introduction

Despite the power of randomized clinical trials (RCTs) to test the effectiveness of treatments, they are not without risk for their participants and for medical research as a whole. RCTs are risky because they remove decision-making power from the patient and her physician, and may require patients to receive a treatment that is ultimately found to be inferior to its comparator, or to standard care. Moreover, they are expensive in terms of direct and opportunity costs, some of which are ethically relevant to the patient’s autonomy or well-being. Therefore, it is generally accepted that there should be some meaningful standard or standards by which to determine when RCTs are ethically permissible.

Equipoise is very commonly cited as the criterion that determines whether an RCT is ethically permissible and is commonly used as a standard by research ethics boards when assessing the appropriateness of proposed clinical trials [1–4]. However, “equipoise” has been defined in several different ways in the literature [5–7], each of which implies a different criterion for assessing RCTs. In 1973, Charles Fried defined “equipoise” as uncertainty on the part of the enrolling physician [8], and Benjamin Freedman revised the concept in 1987 to refer to “honest professional disagreement” at the level of the medical community [9]. Neither concept has been standardized as the single criterion by which RCTs are ethically evaluated. Moreover, both conceptions have been subject to significant criticism from ethicists [10, 11], trialists [12], and clinicians [13–15]. Other definitions of equipoise have been put forward [16], including defining equipoise as a balance of risks and benefits of treatments [7] or centring the patient-participant’s equipoise as the necessary ethical factor permitting randomization [17]. Empirical attempts to determine how trialists define equipoise revealed that they were “baffled” [18].

A common theme in this criticism relates to the challenge of operationalization, which is the process of turning a concept into a protocol or decision-aide that can be used by clinicians, administrators, or researchers to assess the ethical standing of a given RCT. The problem here is that it is not obvious how to establish whether equipoise (by any definition) is present around a given clinical question. Is it appropriate and necessary for an individual physician to assess his or her own uncertainty prior to enrolling each potential trial participant? Should communities of specialist physicians, generalist physicians, or patients be surveyed to establish whether “honest professional disagreement” exists? Even if such steps were to be taken, how much uncertainty would be sufficient, how should this uncertainty be measured, and whose uncertainty would be most important? It has been suggested that establishing the existence of equipoise involves an assessment of the available medical literature, but uncertainty may exist in the minds of a community of physicians independently of whether it exists at the level of the medical literature, or vice-versa. In this way, different definitions of equipoise may be mutually incompatible when we try to operationalize them. These issues become tangible when physicians experience difficulty communicating about equipoise to potential participants [19].

These many points also illustrate a deeper worry about equipoise: some operationalizations are more permissive than others. If the presence of equipoise is assessed through a casual poll of nonexpert physicians, then it may represent such a low threshold that essentially any RCT becomes permissible. Going in the other direction, a politically or socially controversial therapy may create conditions that fit much more demanding operationalizations of equipoise long past the time when reasonable evidence of therapeutic efficacy (or inefficacy) has been generated. It is no surprise to see that controversies surrounding what equipoise is and whether it exists around a given clinical question have arisen in several medicine sub-fields, including stroke neurology [20–22].

In the case of stroke neurology, controversy arose about a decade ago around the proposal to conduct RCTs comparing endovascular thrombectomy to standard care for acute ischemic stroke, where standard care included intravenous thrombolysis for some patients but not for all. Thrombectomy had largely been adopted as an effective treatment despite RCT evidence to the contrary [23] and was being widely used in routine clinical practice. Therefore, enrolling a patient into a trial had the prospect of seeing that patient be randomized to not receive thrombectomy, a treatment that was considered a standard treatment by many expert physicians. This scenario led some physicians to feel that participation in such a trial would be violating their fiduciary responsibility to their patients, and would therefore be unethical. Interestingly, belief in the superiority of thrombectomy existed despite data from three well-conducted RCTs that had found it to provide no benefit over standard care. Was there, or was there not, uncertainty regarding the relative efficacy of standard care vs. thrombectomy, and was this determination to be based on the opinions of physicians or on the state of the medical evidence? Was there, or was there not, equipoise around the question of acute stroke treatment?

Inspired by problems such as these, this paper attempts to describe the various concepts of equipoise and any associated operationalizations by interviewing different stakeholders in the clinical research enterprise: clinical trialists from the field of stroke neurology, research ethics board chairs, bioethicists, and philosophers of medicine. We are not aware of a previous study that has sought to achieve this goal, and our goal was to capture the opinions of a broad range of stakeholders who likely had been forced to develop a concept of equipoise that was applicable to clinical research.

## Methods

### Research design

This study utilized a descriptive questionnaire to capture the opinions of respondents. The questionnaire itself was delivered via a series of structured interviews with the option for the interviewer to ask further questions to clarify any points of confusion. Upon capturing the data, we used qualitative thematic analysis to analyse participant responses. Data are presented as themes, and as descriptive percentages of responses.

### Questionnaire design

We conducted a series of interviews with stakeholders about various problems in RCT ethics (Appendix A). This study was approved by the Ottawa Health Sciences Network Research Ethics Board. Interviews consisted of 10 questions asked of all participants, though the interviewer followed up on interviewees’ individual responses when further exposition appeared relevant. Of the 10 standardized questions asked of all participants, 4 explicitly related to equipoise; those results are reported here. Participants were asked to define “equipoise”, to suggest how it might be operationalized, whether they felt it was a helpful concept to use when evaluating RCTs, and whether they had ever experienced difficulties with using it. When it came to operationalizing equipoise, participants were offered the opportunity to expand on their comments by asking them to think about how they would determine whether equipoise exists around any given trial, and what they would do to assess this.

The respondents were also asked to provide feedback at the end of the interview on anything that was not covered by individual questions. Additionally, members of the research ethics board group were asked to quantify the number of protocols they reviewed annually and describe their processes for doing so.

### Participant selection

We identified chairs of research ethics boards (REBs), representatives of government regulatory agencies, as well as leaders in stroke research, and philosophers (of science, bioethicists, medicine, or ethics). We aimed to obtain 50 respondents, out of feasibility and convenience: 15 clinical investigators, 15 philosophers, 15 chairs of research ethics boards/institutional review boards (REB Chairs), and 5 members of government regulatory bodies. Interview participants were identified based on listed contacts on national regulator and REB websites, university websites, and through MS’s contacts within the academic stroke community. A list was compiled of potential participants, and invitations were sent based on available contact information. Participants were only interviewed once. Stroke researchers were chosen to represent the research community and given access to this population due to the authors’ contacts, and because, as detailed above, a debate over equipoise had recently occurred in this community. Email invitations were sent, and interviewees signed and returned consent documents. Participants were offered a $100 CAD honorarium for their time.

### Data collection

In accordance with the processes laid out by Braun et al. [24] and Pope et al. [25], interviews were done over the telephone, digitally recorded, and transcribed. All interviews were performed by MS, a male physician trained in qualitative research.

### Data analysis

Transcriptions were performed by two team members (SC and BD). After transcription, interviews were coded by BD and MS using a directed thematic approach, using categories and themes grounded in their knowledge of the field of research ethics. Themes were generated from a simultaneous systematic review of the literature on reasons for permitting clinical trials and included categories, such as “Individual MD Uncertainty”, “MD Community Uncertainty”, “Evidence-Based Uncertainty”, that overlapped with known definitions of equipoise. These foundational themes were then joined with those themes identified by utilizing a process described by Gagliardi et al., wherein responses were first sorted into themes (first-level coding), and then these themes were either expanded, focused or merged (second-level coding). If a respondent offered more than one codable response, the response that the participant seemed to favour was coded. Where respondents offered multiple responses to a question, we have endeavoured to discuss them in the text. We then performed simple descriptive statistical analyses within Microsoft Excel on the themes for comparison between and among groups of stakeholders.

## Results

Interviews were completed with 45 participants (Table 1) between October 4, 2016, and April 8, 2019. To obtain the desired number of responses, invitations were sent to 61 clinical investigators (response rate 24.5%), 84 REB Chairs (response rate 17.8%) and 33 philosophers (response rate 45.4%). Participants were primarily located in Canada and the USA (43/45). Despite repeated attempts to contact members of regulatory agencies including the Food and Drug Administration and Health Canada, no employee of these agencies agreed to participate. Demographics of the respondent groups are presented in Table 1. The average interview length was 26:38, with the longest interview being 51:11 and the shortest being 13:38.

**Table 1:** Participant demographics

	**Clinical researchers**	**Philosophers of science**	**REB Chairs**
Age			
30–49	10 (66%)	4 (27%)	5 (33%)
50–69	4 (27%)	10 (66%)	10 (67%)
70 +	1 (7%)	1 (7%)	0 (0%)
Gender			
Male	10 (67%)	10 (67%)	6 (40%)
Female	5 (33%)	5 (33%)	9 (60%)

### “How do you define equipoise?”

Respondents defined the concept of equipoise in many different ways, which were sorted into 7 themes (Table 2). Notably, 2 respondents could not define the concept at all. The most common definition, offered by 14 respondents (31%), related equipoise to disagreement at the level of a community of physicians. The majority (11/14, 79%) of respondents providing this definition were philosophers. The most common definition offered by both investigators and REB chairs related equipoise to an examination of the extant literature, what we have termed evidence-based uncertainty (see Table 2). There was thus heterogeneity in definitions offered between and within groups. Philosophers had the least heterogeneity, defining it three ways, while investigators defined equipoise five ways, and REB Chairs defined it seven ways.

**Table 2:** Definitions

Coded as:	Defined as:	Number of PI respondents	Number of REB responses	Number of philosopher of science responses	Total responses	Relative frequency	Representative quote
MD community uncertainty	Benjamin Freedman’s classic conceptualization of equipoise as disagreement in the physician community	2	1	11	14	31.11%	So, if we're talking about clinical equipoise, then, it's about there being uncertainty in the expert community – Phil 10
Evidence-based uncertainty	This is a demonstrated difference of opinion — typically established through a systematic review of the literature	5	4	2	11	24.44%	Equipoise, to me, means that in the scientific landscape, in the scientific literature, the treatment has not been determined to be beneficial or not beneficial to an individual with a particular condition – PI 2
Uncertainty not otherwise specified	A statement that ‘there must be uncertainty’, without specification of whose uncertainty or how it should be established	4	3	0	7	15.56%	For me it means that there is a level of uncertainty in the decision making process so that you don't know something or that there is just a reasonable level of indecision or lack of clarity about the best method of action – PI 8
Risk vs. benefit	Equipoise as a balancing of health benefits vs side effects or research burdens for the patients	1	2	2	5	11.11%	Well, you’re balancing the risks and benefits of the study, basically. The risk to the patient and the benefit for the gain of the knowledge. – REB 7
Individual MD uncertainty	Uncertainty of one physician making a decision about whether the trial is justified or if a particular patient is a viable candidate for a trial	3	0	0	3	6.67%	I view it as the concept that with respect to the trial that if it’s equally likely in my mind, based on all the data that exist or don’t exist, that these two treatments could be equal or one better than the other, and there’s nothing pointing me to say which one is better than the other, then I think that in my mind, I have equipoise. – PI 4
No/unknown definition	No definition offered	0	2	0	2	4.44%	Well, equipoise, I think is a term, just sort of invoked to a large extent in order to justify something – REB 5
Honest null hypothesis	Equipoise exists when the groups are the same	0	1	0	1	2.22%	So, clinical equipoise to me means that, given everything that I know about these two arms, the outcomes should be the same. That this is not an inferiority design, that we don't think one will be worse than the other, it's really based that they are the same – REB 11
Equal opportunity	This definition is unclear	0	1	0	1	2.22%	That they are giving equal opportunities to every possibility – REB 10
Inaudible	Inaudible	0	1	0	1	2.22%	
	Total responses:	15	15	15		100.00%	

### “How do you operationalize equipoise?”

Respondents provided multiple ways of operationalizing equipoise, which were also sorted into 7 themes (Table 3). Two respondents believed that equipoise could not be defined so that it could guide decision-making, and one REB chair was unaware of the concept and offered no opinion on how it might be put into practice. The most common operationalization of equipoise was to link determinations of uncertainty to some form of literature review, either through an informal review of existing data or through a systematic review and meta-analysis (15/45, 33%). However, these responses exclusively came from researchers and philosophers. However, 6 respondents from the research ethics board group did note that some form of literature review was an important tool to be used in conjunction with other methods of operationalizing equipoise.

**Table 3:** Operationalizations

Coded as:	Defined as:	Number of PI respondents	Number of REB responses	Number of philosopher of science responses	Total responses	Relative frequency	Representative quote
Literature review	Equipoise is established through a literature review, either informal or through systematic review and meta-analysis	7	0	8	15	33.33%	We need a really good systematic of all the literature out there, we have to actually define the terms of who is the scientific community instead of just pointing to the scientific community and saying ‘they are uncertain’, so let’s actually define the parameters. – Phil 6
REB expert opinion	Equipoise is established through the opinion of physician experts, either on the REB on contracted externally to the REB	0	5	3	8	17.78%	Well, I think it has to be sort of a common–sense clinical judgment, I mean, I think we ask the physicians who have a sense of the safety, a sense of the alternative treatments, a sense of what people need, whether a control arm or an alternative, well all these different arms, whether it should be clinically obvious or accepted that one rather is better or worse than the others. – REB 15
Investigator claim	Equipoise is established as a result of belief in the PI and his/her arguments	0	6	0	6	13.33%	Yes, and it helps to have trust in the researcher. If it’s somebody that you know and that you work well with and that has come often to consult, it’s easier than for somebody you don’t know and you’re evaluating for a researcher that’s outside of the institution. That’s when I would need to be more sure of myself – REB 6
Asking colleagues	Equipoise is established through asking peers, either informally or through a formal process like surveys	5	0	0	5	11.11%	[We] establish equipoise by surveying the key stakeholders in that particular area of research before even embarking on any sort of retrospective observational study or prospective observational study to get a sense of whether or not there is any equipoise in the frontline workers at all. – PI 6
REB vote	Equipoise is established through consensus of REB members	0	2	2	4	8.89%	I’m happy that we really debate it and discuss it before we will accept it as a clinical trial. And we need to all feel very comfortable to eb able to say ‘if my patient were randomized to either arm, we would feel okay with that’. – REB 11
Making an argument	Equipoise is established through convincing the REB that it exists	3	0	0	3	6.67%	Well, I think it’s like any scenario, you’re making an argument. Your thesis is there is equipoise and then you have to go and convince people that there is. – PI 5
Can’t be established	Equipoise cannot be established	0	0	2	2	4.44%	Well, I really think would like to put that question to the proponents of equipoise, who I think have done an extremely poor job of defining it – Phil 5
Individual risk assessment	An individual member of the REB determining whether there is a balance of risk/benefits to patients participating in this study	0	1	0	1	2.22%	That’s a tough question. Basically, we just look at it and see whether the risks involved with it appear to us to be worth it. I don’t know, I can’t do more than that. – REB 7
No answer		0	1	0	1	2.22%	
	Total responses:	15	15	15	45	100.00%	

The remaining operationalizations of equipoise are related to assessments of the group or individual beliefs. Two REB Chairs and two philosophers said that equipoise could be determined through a vote of the REB: if the membership of the REB felt that there was equipoise, then equipoise existed. Similarly, 5/15 investigators said that equipoise could be determined by asking peers, either informally or through a formal surveying process, to determine if uncertainty existed around a specific research question. However, no respondent offered a threshold for disagreement or uncertainty necessary to establish equipoise through either the REB vote or the survey.

Among REB chairs, the majority identified the opinions of individual experts and researchers as essential to operationalizing equipoise, be it through the opinions of the study principal investigators, internal experts, or external experts. Some investigators similarly identified that it was their role to convince the REB of the existence of equipoise.

### “Do you find the concept of equipoise helpful?”

**Fig. 1:**
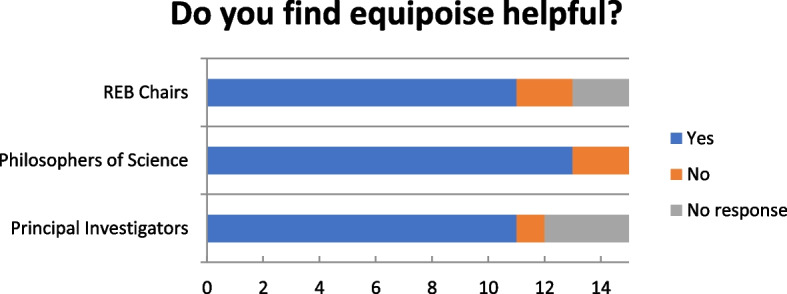
“Do you find equipoise helpful?”

The majority of respondents in all three groups indicated that they felt that the concept of equipoise was helpful (Fig. 1), though there were observable differences in how strongly equipoise was endorsed — from the ardent, “number one condition” and “a guiding principle” to the moderate, “helpful as an ideal” to less enthusiastic, “I would say yes, but it’s really because that’s what I’ve been taught”. Furthermore, respondents who did not find equipoise helpful argued that it was unhelpful because of difficulties involving definition and operationalization. Additionally, respondents proposed that equipoise’s usefulness may be context-dependent, drawing a distinction between its use at the regulatory level vs at the bedside. For example, a philosopher-clinician noted, “when I’m there in a clinic with a patient, it’s embarrassing to say, but [equipoise] doesn't enter my mind because I trust it's been dealt with”.

### “Have you ever had troubles with the concept of equipoise?”

**Fig. 2:**
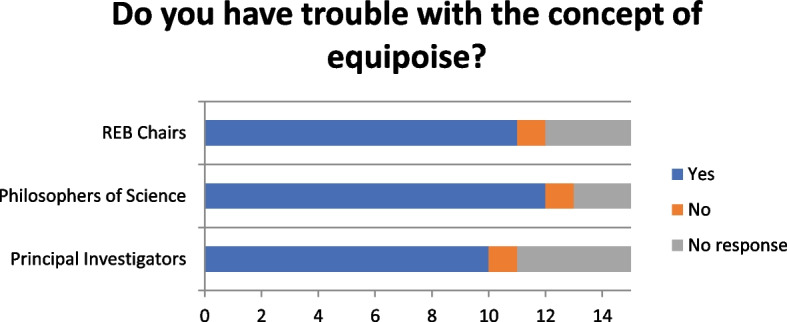
“Do you have trouble with the concept of equipoise?”

The majority of respondents reported having had difficulty applying the concept (Fig. 2), largely due to difficulties with definition. As described by one philosopher, it is a “vague concept”; another noted that “I think that perhaps, one crux of the problem is the fact that there are variations in what equipoise means depending on the situation and what we’re talking about”. Another investigator worried that “you don’t know what’s influencing your decision on whether there’s equipoise”. Particularly challenging circumstances included “very new ideas”, for example where there might be limited or “missing data” such that the “advantage or disadvantage” of a new drug was difficult to know a priori. Conversely, too much data also presented a challenge to equipoise as one philosopher noted that “there’s now so much data that no one person can be responsible for managing all that”. Another philosopher noted that a “very broad range of evidence bases and types of uncertainty” fall under equipoise’s umbrella, further complicating equipoise’s implementation.

## Discussion

This series of interviews with 45 stakeholders demonstrates that there is meaningful heterogeneity in how equipoise is defined and how it might be operationalized. While equipoise was offered to have multiple different definitions by the participants of this study, the vast majority of these definitions (77.8%) relate equipoise to some form of uncertainty. This finding reflects the existing literature and the results of our systematic review [16]. Moreover, the forms of uncertainty that participants related to equipoise seem to be empirically assessable in one way or another; for example, by reviewing the available literature or by surveying experts in the field. This finding is important, in relation to the fact that systematic reviews appear to be rarely done in the lead-up to RCTs and surveys of physician opinion remain an underdeveloped tool to assess the presence of community disagreement [26]. These techniques suggest a way forward to improve the utility of equipoise as a concept that can differentiate between permissible and impermissible RCTs, though much work needs to be done to standardize any such operationalizations. It is therefore perhaps unsurprising that while respondents felt equipoise could be helpful they also struggled with its definition and operationalization.

The findings of this study support the conclusion that (a) stakeholders endorse equipoise “as a standard”, but (b) stakeholders do not agree upon what definition of equipoise consists of that standard, and (c) stakeholders do not agree about what kind of real-world test would be indicative of the presence or absence of equipoise. These conclusions are largely in line with prior similar qualitative work [18, 19] and a large literature critical of equipoise [10–15].

That said, it is possible that the absence of a single standard concept of equipoise linked to an established protocol for determining whether it exists in relation to some clinical question may not be a problem for clinical research. If, for example, all of the available definitions guarantee the necessary uncertainty to justify conducting an RCT, then non-uniformity would be interesting but not problematic. It is possible to imagine a circumstance in which an individual physician is uncertain about which treatment is beneficial, and that there is also a significant split among a group of physicians regarding the same question. However, there is no guarantee that this is ever the case, nor is there a standard system to assess whether it is. Moreover, defining equipoise in terms of the uncertainty of a given investigator or REB, in contrast to the whole of medical knowledge, are very different standards by which to render a given RCT permissible. It is very easy to imagine a trial meeting one of the definitions of equipoise — say, the uncertainty of an investigator — and yet being demonstrably redundant or even harmful when contextualized in relation to all prior research. To say that a matter of such importance — whether patients should be entered into an expensive and potentially risky RCT — can be adequately answered by the opinion of any one physician, or one panel of reviewers, appears inadequate. The culture of modern medical research generally recognizes the superiority of systematic literature reviews over individual physician opinion or group consensus for answering questions about treatment efficacy [27],and yet only 24% of respondents defined equipoise in relation to the state of medical literature.

These conclusions matter because both non-uniformity in the definition of a concept like equipoise as well as the lack of a clearly accepted, standardized operationalization, suggest that current practices may expose patients to previously unrecognized ethical risks. First, there is a risk posed by a presumption of univocality among researchers or trialists, meaning that two members of the research community may mean different things when using the same word, but not recognize that state-of-affairs, and thus not take steps to address disagreements that could arise if they had recognized they were talking about different things.

Concern surrounding the clarity of a term like “equipoise” compound to existing concerns surrounding transparency in the practice of research ethics oversight. Prior work suggests that the fundamental ethical principles underlying RCTs are rarely discussed nor made explicit in relation to individual trials.^35^ So, if an investigator using the word “equipoise” to an REB and simply means his own uncertainty but an REB presumes that a literature review has been completed, then the REB will have been misled about the scientific merit of a potential RCT. The same problem could arise in patient-facing communications if patients understand the concept one way and her physicians are using it in another.

This lack of consistency creates a risk of potential unfairness. It is possible that an REB in one centre considers equipoise to have been met using a weak criterion like individual physician uncertainty, which may be an unacceptable criterion at other centres. It may be unfair for some patients to be exposed to risk or more risk because of this non-uniformity, or to be deprived of access to a promising treatment on the same basis.

Finally, there is the risk of self-dealing, in that the lack of consistency surrounding equipoise allows for the development of potential conflicts of interest. Several REB Chairs reported relying, either in whole or in part, on the attestation of a trial’s principal investigator about whether the equipoise criterion has been met around the trial that the investigator is proposing. If equipoise is to be determined by REBs, who rely upon the attestations of investigators seeking REB approval to determine whether equipoise exists, then the requirement to achieve equipoise becomes at best circular if not overtly conflicted. As 20% of our respondents implied, convincing a REB of equipoise’s existence seems like just another hurdle to overcome before getting a trial off the ground rather than an important guardrail that protects patients from potentially fatal harm, let alone protecting against medically unnecessary research.

## Limitations

This study relies on responses from 45 participants, and thus our ability to generalize from this sample is limited. Researchers were recruited from a related set of disciplines (stroke neurology, cardiology, thrombosis, etc.) and so their opinions may not be representative of views of researchers in other branches of medicine. The selection of these disciplines was out of convenience based on our familiarity with the field, and given the widely recognized controversies in this field we felt that they would be attuned to the issues we were exploring. However, this series of interviews was relatively robust compared to similar published studies [18]. Because some clinician-researchers were identified through the research team’s existing contacts, we may have attracted like-minded individuals. However, given the diversity of opinions offered for each of the questions about equipoise, it seems doubtful that this was the case. Finally, we failed to capture the opinions of regulators, which had been a prespecified goal of this project. We also did not seek to capture the opinions of research funders. We remain interested in exploring their views in future work.

Interviews, while containing the four standardized questions, were open-ended and heavily dependent on the individual researcher conducting the interviews (MS). As such, potential biases may have been introduced through the previously developed beliefs of the interviewer, but a script was followed to mitigate this effect and respondents were not told anything about the hypotheses or aims of the interviews until after they were completed. Additionally, having had one interviewer conduct all interviews helped to minimize stylistic differences between interviews. The interviewer sought to follow up on responses in order to maximize clarity and specificity. While questions were asked in a standard fashion, we cannot eliminate the possibility that they were understood differently by respondents. Additionally, other respondents were selected because they had been identified as influential scholars in this area and were expected to have relevant insights. However, the issues explored in these interviews were not discipline-specific.

## Future directions

As is implied by the perceived importance of the concept of equipoise, further research — both descriptive and normative — should be undertaken to both better understand how equipoise is put into practice, and how it *should* (if at all) be put into practice. The questions we asked our participants about equipoise were a subset of a larger questionnaire, and we intend to analyse respondents’ general understanding of RCT ethics and approval processes in subsequent analyses. Assessing opinions from stakeholders is but one way of cataloguing how the medical community approaches RCT justification, and equipoise specifically. We have sought to analyse the state of the extant literature on this front as well [16]. We are also developing an alternative framework for understanding the ethical and epistemic standards that should be applied to trials based on their epistemic contexts [28], and are interested in developing this approach further. Depending on the results of these various projects, we may ultimately conclude that equipoise should be replaced with a different ethical framework that may be more beneficial for researchers, regulators and trial participants [29, 30].

## Supplementary Information

**Additional file 1:** **Appendix A.**

## Data Availability

Data used for this project will be available upon reasonable request.
