# Supplementary material for: What do we talk about when we talk about “equipoise”? Stakeholder interviews assessing the use of equipoise in clinical research ethics
Source: Trials. 2023 Mar 18;24:203. doi: 10.1186/s13063-023-07221-3 (PMC10024829; doi:10.1186/s13063-023-07221-3)
Supplement: Supplementary file 1 — Additional file 1: Appendix A. [file 13063_2023_7221_MOESM1_ESM.docx]

1. **Template for Semi-Structured Interviews (Researchers & Philosophers)**

Category: [ ] Neurovascular [ ] Cardiovascular [ ] Bioethics / Philosophy

Sex: [ ] F [ ] M

Age: [ ] 30-49 [ ] 50-69 [ ] 70+

Introduction: Thank you for agreeing to this interview. I do not intend to take more than 30 minutes of your time. I would like to ask you a few questions about the ethics of randomized clinical trials (RCTs), to gather your personal experience from a practical perspective, and your thoughts on some of the theoretical issues at play.

1. Have you ever enrolled patients into a randomized clinical trial? How often do you enroll patients in to RCTs?
2. Have you ever had an experience where enrolling patients into a RCT made you uncomfortable? If yes, when and why?
3. In your opinion, is it ethically justifiable to enroll patients into RCTs? Why or why not?
4. Are there circumstances under which it might be unethical to enroll patients into RCTs?
5. The term “equipoise” is often used in reference to the ethics of RCTs. What does “equipoise” mean to you? Do you find this concept is helpful in determining whether to enroll patients into RCTs? Have you had any troubles applying the concept of equipoise? How would you establish whether “equipoise” exists around a clinical trial?
6. The term “fallibility” has been suggested as an alternative concept to “equipoise.” What does “fallibility” mean to you? Do you think fallibility may be helpful in determining whether to enroll patients into RCTs?
7. What is your understanding of the system for ethical review of RCTs in your jurisdiction and at your institution? Do you think this system functions well? Would you like to change it in any way?
8. Do you feel that RCT regulators at the national or local level have sufficient scientific expertise to appreciate the scientific and ethical complexities involved in RCT enrollment?
9. Thinking about the conversation we’ve had, can you sum up in your own words: Under what conditions – ethical, scientific regulatory – are RCTs appropriate and necessary?
10. **Template for Semi-Structured Interviews (REBs and Regulators)**

Category: [ ] Research Ethics Board [ ] National Regulator

Country: [ ] Canada [ ] USA [ ] Great Britain [ ] Europe

Sex: [ ] F [ ] M

Age: [ ] 30-49 [ ] 50-69 [ ] 70+

Introduction: Thank you for agreeing to this interview. I do not intend to take more than 30 minutes of your time. I would like to ask you a few questions about the ethics of randomized clinical trials (RCTs), to gather your personal experience from a practical perspective, and your thoughts on some of the theoretical issues at play.

1. How many RCT applications do you receive each year (on average)? How many are approved (on average)?
2. How many RCT applications did you receive in 2015? How many were approved in 2015?
3. How often do you require protocol amendments or adjustments?
4. What proportion of protocols are rejected?
5. What is your process for RCT review?
6. Does your review process include someone with specialized knowledge in that field of study (for example, a stroke researcher for a study about stroke treatment)?
7. Do you ever request external expertise to review RCT protocols? If so, how often? How do you identify these individuals?
8. The term “equipoise” is often used in reference to the ethics of RCTs. What does “equipoise” mean to you? Do you find this concept is helpful in determining whether RCTs are ethical? How would you establish whether “equipoise” exists around a clinical trial? Have you had any troubles applying the concept of equipoise?
9. The term “fallibility” has been suggested as an alternative concept to “equipoise.” What does “fallibility” mean to you? Do you think fallibility may be helpful in determining whether to enroll patients into RCTs?
10. Do you feel the process by which RCTs are evaluated at your institution is appropriate? Would you like to change it in any way?
11. Are you aware of any RCTs being conducted that you think have ethical problems?
12. Do you feel that RCT regulators at the national or local level have sufficient scientific expertise to appreciate the scientific and ethical complexities involved in RCT enrollment?
13. Thinking about the conversation we’ve had, can you sum up in your own words: Under what conditions – ethical, scientific regulatory – are RCTs appropriate and necessary?
